# Genetic analysis of *Caenorhabditis elegans* Haspin-like genes shows that *hasp-1* plays multiple roles in the germline

**DOI:** 10.1242/bio.059277

**Published:** 2022-07-07

**Authors:** Jommel Macaraeg, Isaac Reinhard, Matthew Ward, Danielle Carmeci, Madison Stanaway, Amy Moore, Ethan Hagmann, Katherine Brown, David J. Wynne

**Affiliations:** Department of Biology, University of Portland, 5000 N Willamette Blvd, Portland, OR 97203, USA

**Keywords:** Chromosome segregation, Inner centromere, Germline, Meiosis, Spermatogenesis, Oogenesis

## Abstract

Haspin is a histone kinase that promotes error-free chromosome segregation by recruiting the chromosomal passenger complex (CPC) to mitotic and meiotic chromosomes. Haspin remains less well studied than other M-phase kinases, and the models explaining Haspin function have been developed primarily in mitotic cells. Here, we generate strains containing new conditional or nonsense mutations in the *Caenorhabditis elegans* Haspin homologs *hasp-1* and *hasp-2* and characterize their phenotypes. We show that *hasp-1* is responsible for all predicted functions of Haspin and that loss of function of *hasp-1* using classical and conditional alleles produces defects in germline stem cell proliferation and spermatogenesis, and confirms its role in oocyte meiosis. Genetic analysis suggests that *hasp-1* acts downstream of the Polo-like kinase *plk-2* and shows synthetic interactions between *hasp-1* and two genes expected to promote recruitment of the CPC by a parallel pathway that depends on the kinase Bub1. This work adds to the growing understanding of Haspin function by characterizing a variety of roles in an intact animal.

## Abstract (precis)

**Summary:** New mutations in the *C. elegans* homologs of the histone kinase Haspin show roles in spermatogenesis and germline proliferation and genetic interactions with mutations in related genes.

## INTRODUCTION

Haspin is a protein kinase that is important for the correct segregation of chromosomes in mitosis and meiosis (Higgins, 2010; Hindriksen et al., 2017). It is one of many kinases that coordinate the events of M phase, such as Cyclin-dependent kinase 1 (Cdk1), Polo-like kinase 1 (Plk1) and Aurora B. Intricate regulatory interactions have been identified among these proteins, so a complete understanding of this network requires specific manipulations and benefits from being studied using diverse model systems. Haspin inhibitors hold the potential of being effective chemotherapy drugs because features of Haspin's kinase domain are unique among eukaryotic protein kinases (Dominguez-Brauer et al., 2015; Eswaran et al., 2009; Villa et al., 2009). Haspin phosphorylates Histone H3 on threonine 3 (H3T3), which is generally thought to be the single most important substrate for Haspin function during mitosis and meiosis (Dai et al., 2005). The possibility of a single critical target has made Haspin inhibitors attractive chemotherapeutics because of the potential for fewer off-target effects (Amoussou et al., 2018). However, there is some evidence that Haspin may have other targets, including from proteomic studies with Haspin inhibitor treatments, which suggested that the histone variant macroH2A and the centromeric protein CENP-C may be direct targets (Maiolica et al., 2014). The testis-specific histone H2A protein TH2A has been shown to be phosphorylated by Haspin *in vivo*, and the cohesion regulator WAPL is phosphorylated by Haspin *in vitro* (Hada et al., 2017; Liang et al., 2018). The challenge of determining the direct targets of a kinase and the discrepancies that can occur between *in vitro* and *in vivo* kinase activities warrant additional analysis of Haspin function using diverse *in vivo* systems.

Haspin has two overlapping functions in mitosis at the inner centromere, a distinct region of mitotic chromosomes in between the sister centromeres. Haspin helps protect chromosome cohesion by binding to the cohesion-associated protein Pds5 and inhibiting the cohesin-removing protein WAPL (Dai et al., 2006; Goto et al., 2017; Liang et al., 2018; Yamagishi et al., 2010; Zhou et al., 2017). Haspin-dependent phosphorylation of H3T3 (H3T3ph) directly recruits the chromosomal passenger complex (CPC), which plays a variety of roles that promote error-free chromosome segregation (Carmena et al., 2012). The CPC is composed of four subunits, the Aurora B kinase and three accessory subunits, INCENP, Borealin/Dasra and Survivin, which directly binds H3T3ph (Kelly et al., 2010; Wang et al., 2010; Yamagishi et al., 2010). These two mechanisms help explain the phenotypes seen for Haspin knockdown in mitotic cells, which include misaligned chromosomes, loss of cohesion and spindle defects (Dai and Higgins, 2005; Dai et al., 2006, 2009), but the effects of Haspin deletion in unperturbed mitotic cells are relatively mild (Hadders et al., 2020). Haspin is activated by phosphorylation of multiple sites in its long, disordered amino-terminal domain, which has been shown to be a target of Plk1, Cdk1, Aurora B and autophosphorylation (Ghenoiu et al., 2013; Wang et al., 2011; Zhou et al., 2014). It remains to be seen how important these regulatory mechanisms are in a variety of cell types and whether they are conserved in more distantly related species.

The relatively mild phenotype of Haspin loss in mitosis is due to partially redundant function with the spindle assembly checkpoint kinase Bub1, which promotes CPC localization through a second kinetochore-associated mechanism (Yamagishi et al., 2010). Bub1-dependent phosphorylation of histone H2A recruits shugoshin proteins, which recruit the Borealin/Dasra subunit of the CPC (Tsukahara et al., 2010). There has been uncertainty about the interplay between these two parallel pathways (Hindriksen et al., 2017), but recent work in human tissue culture cells supported the model that both pathways can independently recruit the CPC to chromatin (Hadders et al., 2020). The functional significance of different CPC recruitment mechanisms and therefore the existence of distinguishable pools of CPC is still being investigated. Moreover, work is ongoing to understand the interplay between kinetochore-dependent signaling molecules and cohesion regulators. There is a need for better understanding of the extent to which CPC recruitment and cohesion protection mechanisms are altered in different cell types and in different organisms. Cancer cells have been shown to lose the ability to recruit the CPC preferentially to misaligned chromosomes, so understanding how CPC recruitment mechanisms are altered is important for future cancer treatments (Salimian et al., 2011).

In contrast to mitosis, relatively little is known about Haspin function in meiosis, and somewhat contradictory findings have been reported in different organisms. A Haspin knockout mouse is fertile and shows no obvious developmental phenotypes other than some disordered cells in the testis (Shimada et al., 2016). In contrast, Haspin inhibitors cause defects in spindle pole clustering and failure to complete meiosis I in mouse spermatocytes, as well as incorrect kinetochore-microtubule attachments that lead to aneuploidy (Balboula et al., 2016; Nguyen et al., 2014). Surprisingly, Haspin inhibition also caused a loss of condensin on meiotic chromosomes but no defects in cohesion were seen (Nguyen et al., 2014). The meiotic bivalent is a cruciform structure made up of four chromosomes, and Haspin inhibition clearly disrupted CPC localization on the axes between chromosomes but left kinetochore-proximal pools untouched (Nguyen et al., 2014). This phenotype is very similar to the kinetochore CPC pools seen after mitotic Haspin loss, but over a tenfold larger length scale (Bekier et al., 2015). In *Drosophila*, Haspin has been shown to affect transcription during interphase and in histone segregation in germline stem cells (Fresán et al., 2020; Xie et al., 2015). However, loss of function of Haspin does not cause viability defects or meiotic nondisjunction in *Drosophila*, and Haspin-dependent recruitment of the CPC in meiosis is not as important as it is in mouse spermatocytes (Wang et al., 2021). The discrepancies in Haspin's role in meiosis among animals, as well as its additional functions that can be more easily characterized in some systems, justify further study of the protein's function in model organisms.

*Caenorhabditis elegans* is a powerful system to study chromosome segregation in both mitosis and meiosis because there are many genetic and cytological tools available. Mutant alleles in cell cycle regulatory genes are readily available and easily combined by crosses. Individual hermaphrodites produce over 300 progeny, which makes fecundity measurements robust enough to distinguish small differences, and cell division can be monitored through the transparent bodies and eggs of live animals. The *C. elegans* germline is organized so that nuclei move in one direction as they progress through mitotic stem cell divisions and then meiosis, with spermatogenesis and oogenesis occurring at specific life stages in hermaphrodites (Hubbard and Greenstein, 2005). Finally, the sex determination system, in which XO males are produced by meiotic nondisjunction in XX hermaphrodites, facilitates analysis of meiotic chromosome segregation and can distinguish meiotic defects from mitotic (Hodgkin et al., 1979).

Here, we set out to use *C. elegans* to better understand the function of Haspin-related genes in the context of a whole animal. To our knowledge, only a single previous report has tested the function of Haspin proteins in this organism*.* In *C. elegans*, the mechanisms that regulate differential cohesion loss in meiosis have been rigorously studied. Unlike mouse oocytes, in which the CPC is found on both chromosome axes, the CPC is restricted to the ‘short arm’ of the meiotic bivalent in oocytes, the axis in between the homologous chromosomes, where it is essential for anaphase I chromosome segregation (Kaitna et al., 2002; Rogers et al., 2002). The *C. elegans* Aurora B homolog AIR-2 phosphorylates the meiosis-specific cohesin REC-8, which leads to the selective loss of cohesion between homologs in meiosis I (Ferrandiz et al., 2018; Rogers et al., 2002). The mechanisms that restrict the CPC to this region in *C. elegans* have also been well studied. The nematode-specific protein LAB-1 plays a role analogous to that of shugoshin in other systems by protecting cohesion in meiosis I (de Carvalho et al., 2008). Like shugoshin proteins, LAB-1 recruits a phosphatase that antagonizes phosphorylation, including both H3T3ph and the AIR-2-dependent phosphorylation of H3 (Ferrandiz et al., 2018; Tzur et al., 2012). Recent work described a new model for CPC recruitment in *C. elegans* in which the spatial regulation of the CPC is primarily controlled by signaling from HASP-1 protein that is localized by the meiotic chromosome axis, while temporal regulation of the CPC depends on activity of the Cdk1 homolog, *cdk-1* (Ferrandiz et al., 2018). However, a number of questions about the function of Haspin-related genes in *C. elegans* remain. *C. elegans* has two genes similar to Haspin, *hasp-1* and *hasp-2*, and it has not been tested whether *hasp-2* plays an important role. Previous studies in *C. elegans* focused on oocytes, so it is not clear whether *hasp-1* is important for spermatogenesis. In addition, it is not known whether the Bub1-dependent pathway functions in parallel with *hasp-1* to recruit the CPC during meiosis, or if activation of *hasp-1* by Plk1 homologs is conserved in *C. elegans*.

In this study, we analyzed both classical and conditional mutations in the Haspin-related genes in *C. elegans*, *hasp-1* and *hasp-2*. We found that a deletion mutation in *hasp-1* caused sterility due to a defect in germline stem cell proliferation, and we used a conditional *hasp-1* mutation to show that *hasp-1* acts in spermatogenesis as well as oogenesis. Depletion of *hasp-1* caused meiotic segregation errors and maternal embryonic lethality. Combining *hasp-1* depletion with other mutant backgrounds, we showed that *hasp-1* is likely to function downstream of the Plk1 homolog *plk-2*, which is consistent with it being activated by *plk-2*. In contrast, we found that mutations in Bub1 pathway genes caused strong synthetic interaction phenotypes when combined with *hasp-1* knockdown, suggesting that the parallel activity of the Bub1 pathway is required in *C. elegans* meiosis. Our results confirm that many of the regulatory mechanisms described for Haspin in other systems are conserved in *C. elegans* and that this model system will be useful to further test hypotheses for Haspin function and regulation in a variety of cell types in an intact animal.

## RESULTS

### A deletion mutation in *hasp-1* causes sterility due to a lack of germline stem cell proliferation

**Fig. 1.**
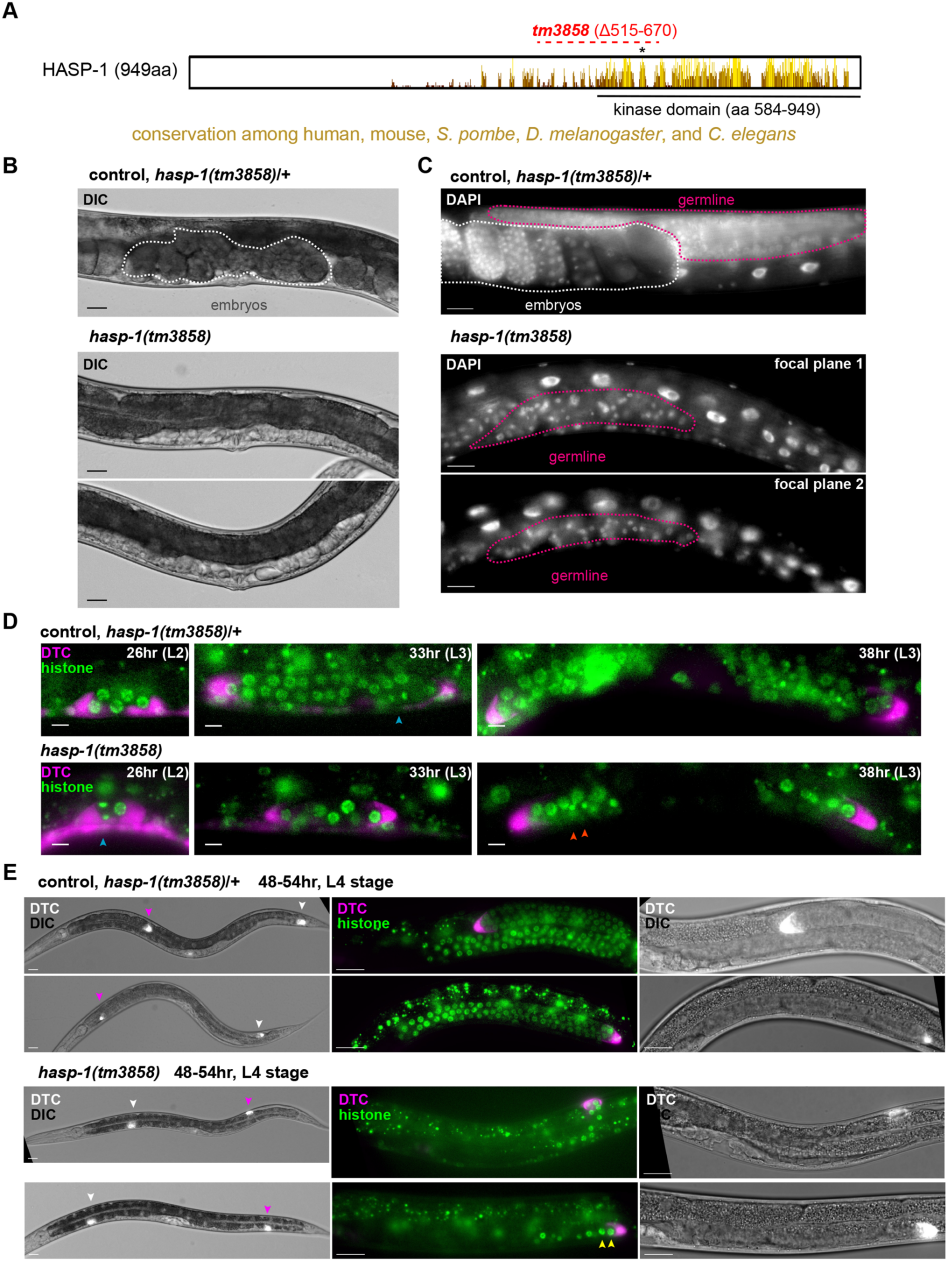
**A putative null mutation in *hasp-1* causes sterility due to a failure of germline stem cell proliferation.** (A) Schematic of *C. elegans* HASP-1 protein, showing locations of conserved residues among human, mouse, *S. pombe* and *D. melanogaster* Haspin homologs. Completely conserved residues are indicated by taller yellow bars, while lower bars with brown shades indicate partial conservation in only some species. Plot prepared using Jalview (Waterhouse et al., 2009). Lysine 644 is shown with an asterisk and the dashed red line indicates the in-frame deletion caused by the *hasp-1(tm3858)* allele. aa, amino acid. (B,C) Nomarski (DIC) and DAPI staining of adult *hasp-1(tm3858)* homozygotes and heterozygous control animals. Embryos are circled (white dotted lines) to highlight the lack of embryos in mutant homozygotes. Two focal planes of the same animal are shown for the homozygote in C to show the lack of germ cells throughout the body (pink dotted lines). Scale bars: 20 μm. (D) Gonads in synchronized larvae (h after egg laying is indicated) visualized using fluorescent markers for nuclei (green) and the distal tip cell (DTC; magenta), which marks the end of the developing gonad arms. Blue arrowheads show nuclei undergoing mitosis; orange arrowheads show small, bright nuclei that may be apoptotic. Scale bars: 5 μm. (E) L4 larvae of the same strain as in D, age matched 48-54 h after egg laying. White and magenta arrowheads mark the two DTCs, and the right two panels are higher-magnification images of the gonad arm marked with the magenta arrowhead in the leftmost panel, oriented with the ventral side down and the vulva to the left. Yellow arrowheads show enlarged nuclei. Scale bars: 20 μm.

The Haspin gene has undergone an unusual expansion in the *Caenorhabditis* lineage, with more than eight predicted proteins that have some homology to the vertebrate Haspin kinase domain (Higgins, 2001). Two of these paralogous genes have the highest homology to human Haspin and have been named *hasp-1* and *hasp-2*. A *hasp-1* deletion allele, *hasp-1(tm3858)* generated by the Japanese National Bioresourse Project for *C. elegans* (Gengyo-Ando and Mitani, 2000), had not yet been characterized to our knowledge. The *hasp-1(tm3858)* mutation is predicted to generate an in-frame deletion of 155 amino acids that include the beginning of the well-conserved kinase domain and a lysine residue (K644) required for kinase activity (Fig. 1A; Villa et al., 2009). We verified the deletion breakpoints (data not shown) and outcrossed the mutation prior to analysis of the phenotype. Hermaphrodites homozygous for *hasp-1(tm3858)* grew to adulthood but were sterile with a slight protruding vulva (Fig. 1B). Sterility and vulval defects are common phenotypes for loss of function of genes involved in mitosis in *C. elegans* (O'Connell et al., 1998). The area around the vulva had a clear appearance due to the failure to produce embryos filling the uterus. We 4′,6-diamidino-2-phenylindole (DAPI) stained whole animals to visualize the gonad and found a dramatic lack of germline nuclei in adult animals (Fig. 1C). We assumed that the lack of germline nuclei was due to a failure of mitotic proliferation of germline stem cells, but we also considered the possibility of defects in somatic gonad development. To better characterize the germline defect, we crossed *hasp-1(tm3858)* into a background containing fluorescent fusion proteins marking the distal tip cell (DTC) and histone H2B, which allowed us to visualize the ends of the gonad arms and the nuclei inside (Fig. 1D,E). During all stages observed, *hasp-1(tm3858)* homozygotes had fewer germline nuclei than controls, with the defect becoming more severe by the late L3 and L4 stages. In mutant animals, some gonads showed chromosomes that appeared to be in anaphase (blue arrowheads, Fig. 1D), but there was only a modest increase in the number of nuclei. In animals 48-54 h after egg laying (which are expected to be in the L4 stage), all DTCs in both mutant and control animals had successfully completed the first phase of migration by moving away from the vulva along the ventral side of the animal. However, the germline stem cell proliferation that normally occurs during DTC migration had not taken place (Fig. 1E). Fewer than ten nuclei were present in mutant gonads and only in the distal region, while control gonads contained over 100 nuclei. Nuclei in mutant gonads also varied in size, with some much larger than in control animals (yellow arrowheads, Fig. 1E). In controls, some DTCs had also completed the later phases of migration by turning and moving back toward the vulva along the dorsal side, but in *hasp-1(tm3858)* animals, DTCs had at most completed only the first turn in migration in this time period. Because the germline proliferation defect was much more severe than the delay in DTC migration, these data support the hypothesis that a defect in proliferation is the cause of sterility in these animals. The lack of germline nuclei in the *hasp-1(tm3858)* mutant led us to use a different approach for further study of *hasp-1* function.

### A nonsense mutation in *hasp-2* has no obvious phenotype

**Fig. 2.**
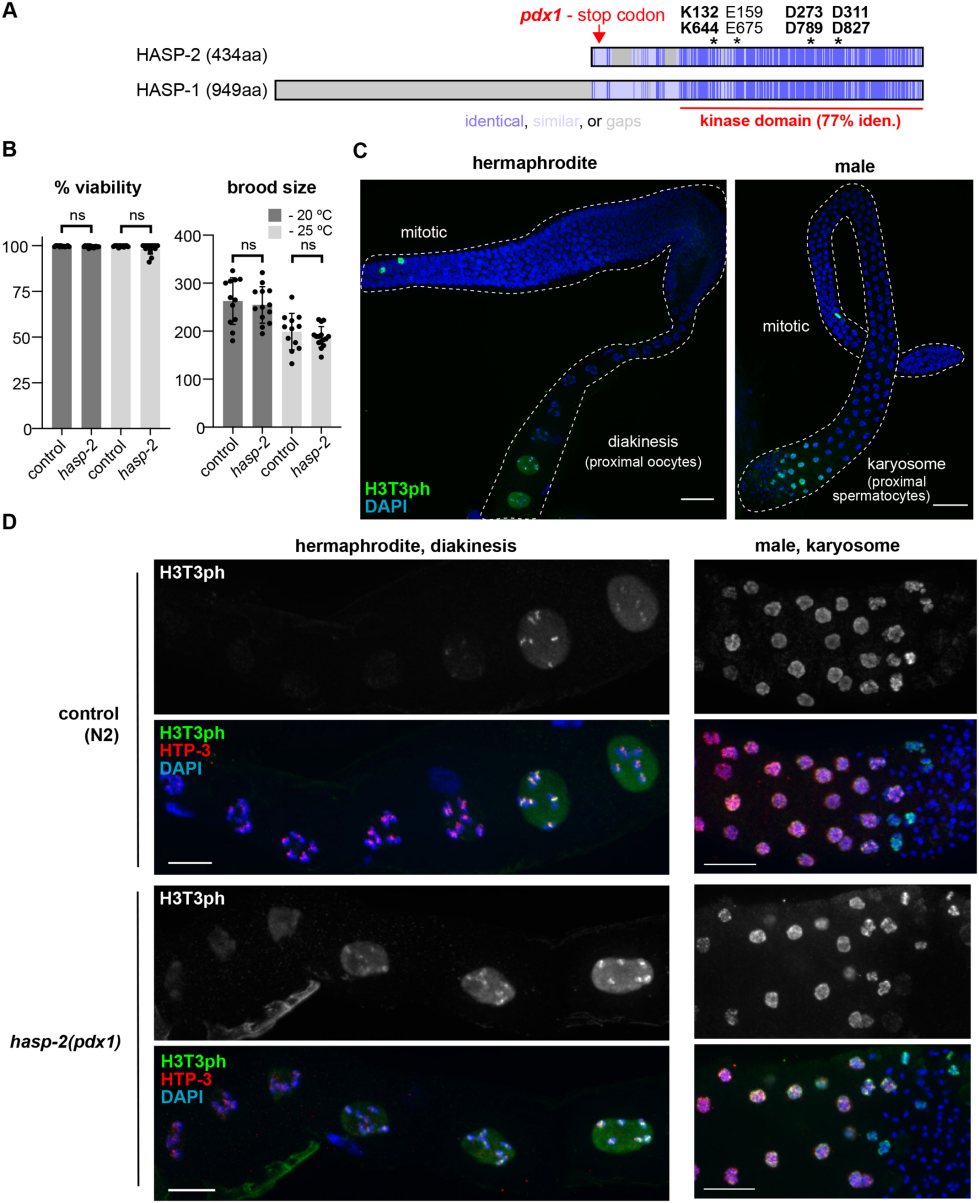
**A *hasp-2* putative null mutation has no effect on brood or H3T3 phosphorylation.** (A) Schematic of HASP-1 and HASP-2 predicted proteins showing locations of conserved sequences (blue) and residues essential for kinase activity (asterisks). aa, amino acid. (B) Average embryonic viability and brood size measurements for whole broods from control (N2) and *hasp-2(pdx1)* animals raised at 20°C or 25°C (error bars show s.d.). ns, *P*>0.05 by Mann–Whitney test. (C) H3T3ph staining in whole dissected gonads from a hermaphrodite (left) and male (right) outlined with dashed white lines. Scale bars: 20 μm. (D) Higher-magnification images as in C, showing the nuclei in the proximal region of the gonad. Scale bars: 10 μm.

The *hasp-1* and *hasp-2* kinase domains have high identity (77%), and residues known to be required for kinase activity are conserved, including lysine and glutamate residues essential for ATPase activity as well as two aspartate residues essential for catalysis and magnesium binding (Manning et al., 2002; Villa et al., 2009). However, the *hasp-2* gene is predicted to produce a much smaller protein than *hasp-1* and to be made up almost entirely of the kinase domain (Fig. 2A). Thus, many of the regulatory mechanisms that have been characterized in the N-terminal portion of Haspin genes in other organisms are unlikely to be shared by *hasp-2*. For example, there are no Cdk1 phosphorylation sites (STP sequences) outside of the *hasp-2* kinase domain, which are expected to activate the kinase (Ghenoiu et al., 2013), and no tyrosine residues, which are conserved in motifs required for Pds5 interaction (Goto et al., 2017). *hasp-1* has been suggested to have a divergent Pds5 interaction motif, but this has not yet been experimentally validated in *C. elegans* (Goto et al., 2017). Based on RNA-sequencing data available through Wormbase, *hasp-2* expression is highest in males and in L4 hermaphrodites, in contrast to *hasp-1*, which peaks in the early embryo. The expression data suggest the possibility that *hasp-2* may have evolved specificity for a role during spermatogenesis. We were interested in this possibility due to the high expression of Haspin in mouse testis relative to other proliferative tissues (Tanaka et al., 1999).

No previously existing mutation in *hasp-2* was available, so we used CRISPR/Cas9 to introduce a small insertion early in the first exon that generated a nonsense mutation followed by a frame shift (Fig. 2A). After verifying and outcrossing the allele, we found that animals homozygous for *hasp-2(pdx1)* had normal embryonic viability and brood sizes at both 20°C and 25°C (Fig. 2B) and no apparent morphological defects. We examined H3T3ph staining in the germline of *hasp2(pdx1)* mutants and saw robust signal in mitotic nuclei and in the proximal gonad regions in both hermaphrodites and males (Fig. 2C,D). Assuming that the *hasp-2(pdx1)* allele is acting as a null mutation, this and the results of double mutant strains discussed below suggest that *hasp-2* does not promote H3T3ph in the *C. elegans* germline. The severe phenotype caused by *hasp-1* loss of function (Fig. 1) and the lack of phenotypes associated with *hasp-2* mutation suggest that *hasp-2* is unlikely to substitute for *hasp-1* activity in *hasp-1* mutant strains, and so we focused our attention on better characterizing the loss of *hasp-1*. We also note that our immunofluorescence experiments did not detect H3T3ph in any other nuclei in the adult hermaphrodite germline, such as mature sperm, suggesting that HASP-1 activity may be limited to meiosis in spermatocytes.

### Conditional degradation of HASP-1 causes maternal-effect embryonic lethality but minimal effects on germline proliferation

**Fig. 3.**
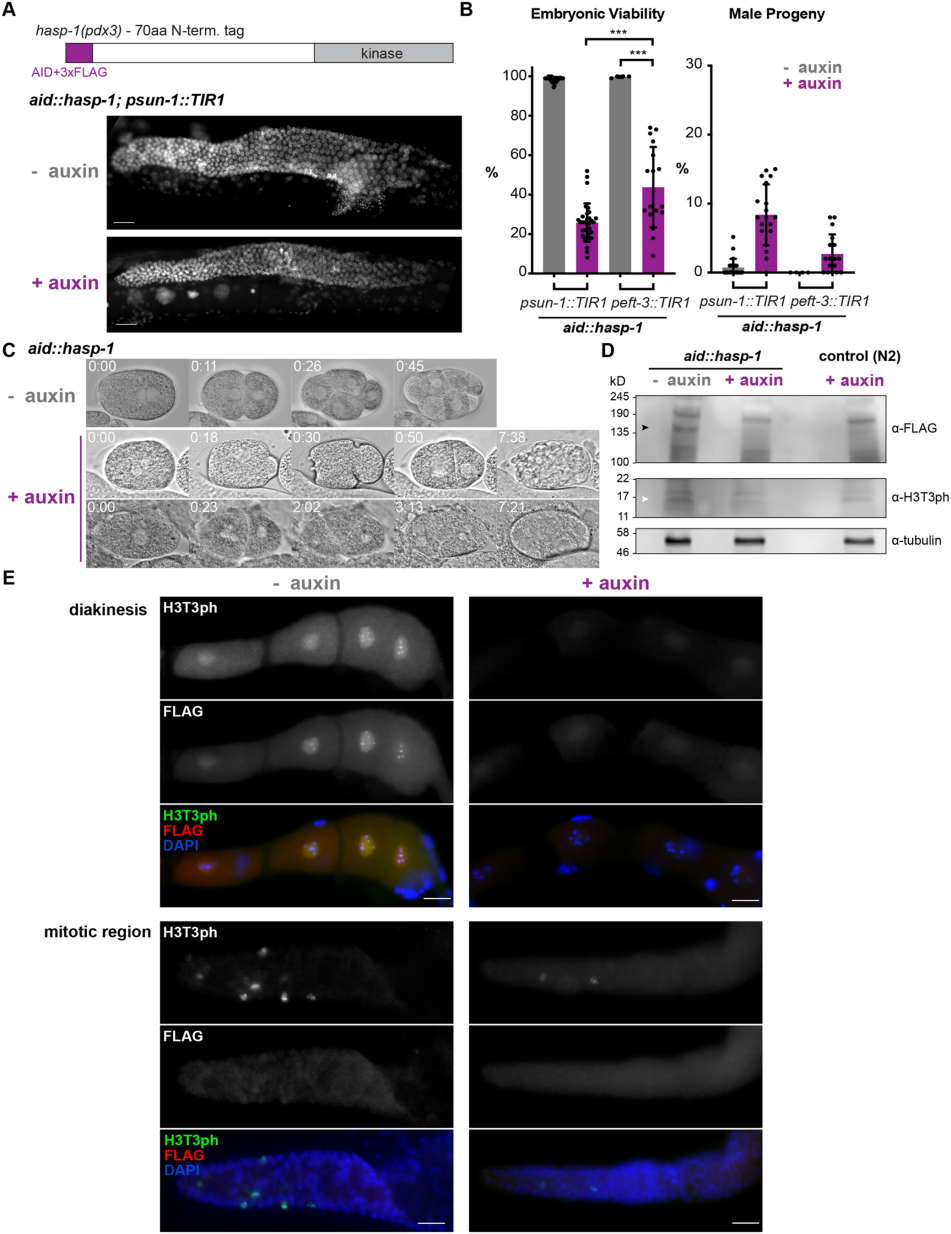
**Analysis of HASP-1 degradation.** (A) Schematic of the *aid::hasp-1* allele (top) and DAPI-stained germlines (below) in whole-mount animals with or without exposure to auxin. aa, amino acid. (B) Average embryonic viability and male production for whole broods (error bars show s.d.) from *aid::hasp-1* animals in which the TIR1 ligase was expressed in the germline (*psun-1::TIR1*) or soma (*peft-3::TIR1*). ***P<0.001 by Mann–Whitney test. (C) Nomarski images of embryos from *aid::hasp-1* animals with or without auxin treatment. Time shown is h:min after pronuclear meeting. (D) Western blots from whole-worm lysates of *aid::hasp-1* animals with or without auxin and control N2 animals. Antibodies against the FLAG epitope recognize a band specific to the *aid::hasp-1* strain (black arrowhead) that is reduced upon auxin treatment, while antibodies recognizing H3T3ph show a partial reduction due to auxin (white arrowhead) but an H3-specific band is less distinct. (E) Immunofluorescence of dissected gonads from *aid::hasp-1* animals with or without auxin treatment. Scale bars: 10 μm.

In order to further investigate the role of *hasp-1* in *C. elegans*, we generated a conditional degradation allele using the auxin-inducible degron (AID) system (Nishimura et al., 2009; Zhang et al., 2015). A previous study generated a *hasp-1* allele with a C-terminal AID tag to characterize *hasp-1* function in oocyte meiosis (Ferrandiz et al., 2018). We independently generated a similar allele, *hasp-1(pdx3)*, but with the AID tag and three FLAG tags on the N-terminus (hereafter referred to as *aid::hasp-1*). Mutant *aid::hasp-1* animals had no obvious phenotype in the absence of auxin, confirming that the tag does not alter protein activity or regulation. We crossed the *aid::hasp-1* allele into a background in which the auxin-activated ubiquitin ligase TIR1 is expressed in the germline under the control of the *sun-1* promoter (Zhang et al., 2015). Unlike the *hasp-1* deletion mutation (Fig. 1), HASP-1 protein degradation in the germline did not cause obvious changes in germline morphology by DAPI staining (Fig. 3A) but did lead to an incompletely penetrant maternal-effect embryonic lethality (25% embryonic viability, Fig. 3B and Table 1). Maternal-effect embryonic lethality can be caused by defects in meiotic chromosome segregation and, consistent with this, the surviving embryos had a high incidence of males (Him) phenotype, which is caused by nondisjunction of the X chromosome in *C. elegans* (Fig. 3B). We monitored the development of embryos from mothers exposed to auxin and saw variable defects in the early cell divisions in embryos that did not hatch. Defects included early mitotic failures that generated abnormally large nuclei, cytokinesis failures or disorganized cleavage furrows producing multinucleate cells, and dead embryos arrested with various cell numbers well prior to gastrulation (Fig. 3C). Together with the Him phenotype, these data suggest that *hasp-1* is required both in meiosis and in the early mitotic divisions in embryos.

**Table 1.**
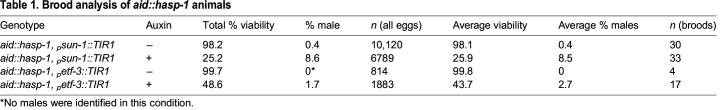
Brood analysis of *aid::hasp-1* animals

Haspin proteins have been shown to play a few roles outside of mitosis and meiosis in other model systems, so we next used our *aid::hasp-1* allele to test whether *hasp-1* is needed outside of the germline. We considered the possibility that the gonad morphology defect seen in the *hasp-1* deletion mutation (Fig. 1) could be due to a requirement for *hasp-1* in somatic tissue during gonad development. We crossed the *aid::hasp-1* mutation into in a background in which TIR1 is expressed under the control of the *eft-3/eef1A.1* promoter and *unc-54* 3′ UTR (*peft-3::TIR1)*, which have been shown to drive expression in somatic tissues but not in the germline (Zhang et al., 2015). We saw no morphological defects after depleting *hasp-1* in this strain: to our surprise, we observed a less penetrant maternal-effect embryonic lethality (44% embryonic viability) and a slight Him phenotype (Fig. 3B and Table 1). Because these phenotypes are similar to those observed upon germline knockdown, and are not expected to result from loss of HASP-1 in somatic tissue, we favor the conclusion that there are low levels of TIR1 protein in the germline in the *peft-3::TIR1* strain that were not previously observed.

Next, we used antibody staining to better characterize *hasp-1* function in the germline. By western blotting of whole-worm lysates, we were able to detect, using anti-FLAG antibodies, a band above 135 kD that was present in *aid::hasp-1* animals (Fig. 3D, black arrowhead) but not N2 controls. This band was reduced when *aid::hasp-1* animals were exposed to auxin for 24 h. H3T3ph antibodies showed high levels of background on blots, but we were able to detect a band below 17 kD that was reduced after auxin treatment in *aid::hasp-1* animals relative to the intensity of the same band in either N2 control animals or non-auxin-treated *aid::hasp-1* animals (Fig. 3D, white arrowhead). To look more directly at the nuclei that should be affected by the tissue-specific depletion of HASP-1, we used immunofluorescence of dissected animals. We could detect faint signal for the FLAG epitope in oocyte nuclei in the proximal region but not in mitotic nuclei in the distal gonad (Fig. 3E). The lack of detectable anti-FLAG signal in mitotic nuclei is surprising because the H3T3ph signal is robust and often covers all chromatin with high intensity, rather than being restricted mostly to the mid-bivalent region as it is in oocytes. The H3T3ph and FLAG signals in oocytes were reduced below detectable levels when *aid::hasp-1* animals were exposed to auxin. There was also a clear reduction in H3T3ph staining in mitotic nuclei; however, some residual H3T3ph could be detected in mitotic nuclei following auxin treatment. Thus, these data confirm that we were able to reduce HASP-1 protein levels using the AID system but showed an unexpected discrepancy between the level of HASP-1 protein detected by the anti-FLAG antibody and the level of H3T3ph.

### Altering the timing of HASP-1 degradation separates phenotypes due to sperm and oocyte meiosis

**Fig. 4.**
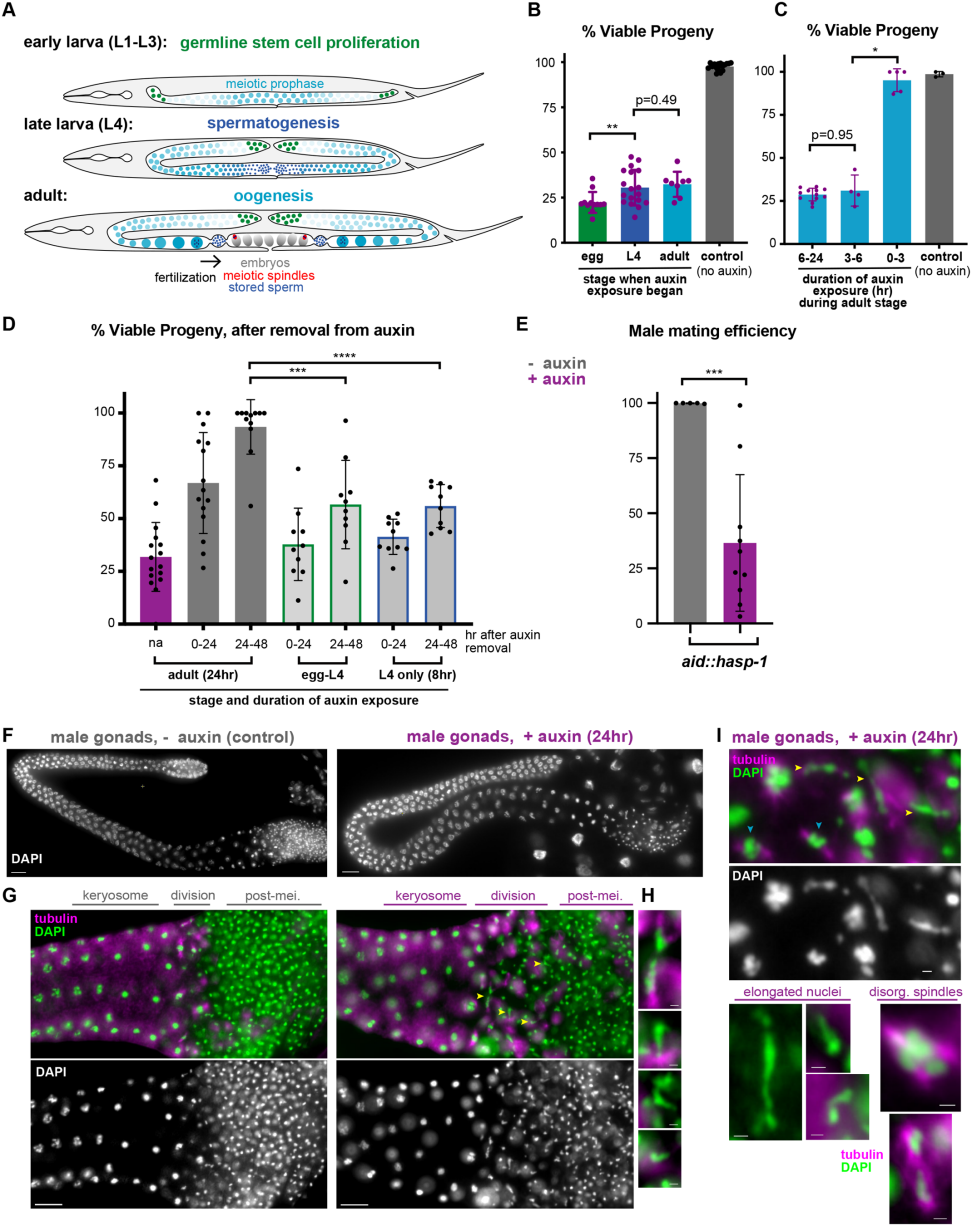
**Loss of *hasp-1* during spermatogenesis reduces progeny viability and male mating efficiency and produces misshapen nuclei in spermatids.** (A) Schematic showing stages of germline development relevant to auxin exposure experiments. (B-D) Average embryonic viability of progeny laid by mothers treated with auxin for various amounts of time. Each data point represents the viability for one plate with 5-20 worms, error bars show s.d. (B) Animals were placed on auxin as eggs, L4s or adults, and the viability of all subsequent progeny was measured. (C) Adult animals were placed on auxin for 3 h (0-3 plates), transferred onto a second set of plates for 3 h (3-6) and then transferred a second time for the remainder of a 24 h period (6-24). (D) Animals were picked onto auxin plates at different life stages and for different amounts of time and then removed onto non-auxin plates for two 24 h periods. For ‘adult’ exposure, adult animals were placed on auxin plates for 24 h (na, not applicable; purple bar, included as a control to verify that treatment produced expected levels of viability based on data in B and C) before two 24 h recovery periods (dark gray bars); for ‘egg-L4’, eggs were placed on auxin and allowed to reach L4 before two 24 h recovery periods (light gray bars, green borders); for ‘L4 only’, L4 animals were exposed to auxin for 8 h before two 24 h recovery periods (light gray bars, blue borders). (E) Mating efficiency of *aid::hasp-1* males crossed to *hasp-1^+^dpy-5 unc-15* hermaphrodites. L4 males and hermaphrodites were transferred to auxin or non-auxin mating plates and allowed to mate for 48 h before males were removed. Each point is the percent of cross progeny (non-Dpy non-Unc) on a single mating plate. ****P<0.0001, ***P<0.001, **P<0.01, *P<0.05 by Mann–Whitney test. (F) DAPI-stained whole dissected gonads from *aid::hasp-1* males treated for 24 h on auxin plates, or untreated controls. Scale bars: 10 μm. (G) Proximal gonad regions of males treated as in F and co-stained with DAPI and anti-tubulin antibodies. Scale bars: 10 μm. (H) Higher-magnification images of elongated DAPI-stained structures corresponding to the yellow arrowheads in G. Scale bars: 1 μm. (I) Additional examples of elongated DAPI bodies and disorganized spindles from males treated for 24 h with auxin as in F-H. Yellow arrowheads and additional examples (below, left) show elongated DAPI bodies. Examples of multipolar and disorganized spindles (below, right) are shown in contrast to some apparently normal spindles (blue arrowheads, above). Scale bars: 1 μm.

Because the maternal-effect, dead-embryo phenotype we observed could be caused exclusively by the known role for *hasp-1* in oocyte meiosis, we next tested whether altering the time of auxin exposure could determine whether *hasp-1* was playing a role at earlier stages in the germline. All *aid::hasp-1* degradation experiments described above were done by transferring mothers to auxin during the L4 stage (when spermatogenesis is occurring), so we reasoned that earlier exposure to auxin could reveal roles in germline stem cell proliferation or spermatogenesis (Fig. 4A). When *aid::hasp-1* embryos were placed on plates containing auxin, they all hatched and grew into fertile adults (30/30), confirming that the embryonic lethality is due to knockdown in the mothers. When mothers were exposed to auxin as embryos, we found only a slight increase in the maternal-effect embryonic lethality relative to mothers exposed during the L4 stage (Fig. 4B). In contrast, exposing mothers to auxin later, after spermatogenesis but during oogenesis, caused similar levels of embryonic lethality as exposure starting during the L4 stage. Together, these data suggest that the most critical time of function of *hasp-1* is during adulthood, presumably during CPC recruitment in oocytes.

Pulse-labeling experiments have shown that oocytes progress from the diakinesis stage to embryos in approximately 10 h (Jaramillo-Lambert et al., 2007), so we tested whether a requirement for *hasp-1* could be seen following shorter exposures to auxin. Remarkably, embryos laid 3-6 h after adult mothers were first exposed to auxin showed similar levels of lethality as embryos laid 6-24 h after exposure (Fig. 4C). In contrast, very low lethality was seen in embryos laid in the first 3 h after exposure.

Since we noticed that mothers exposed to auxin as embryos had lower embryonic viability than those exposed during the L4 stage or later, we reasoned that the difference was due to a role in sperm development, which occurs during the L4 stage in hermaphrodites (Fig. 4A). *C. elegans* hermaphrodites use stored sperm throughout adulthood, so we hypothesized that defects during spermatogenesis would be apparent throughout adulthood even if HASP-1 degradation was no longer occurring. To test this, we exposed hermaphrodites to auxin, removed them from auxin and monitored whether the viability of their progeny recovered. When animals were exposed to auxin as adults (after spermatogenesis is over) for 24 h and then removed from auxin, their progeny recovered viability up to 93.5% in the second day after removal (Fig. 4D). However, when animals were exposed to auxin from eggs and then removed at the end of the L4 stage, their progeny only recovered to 56.7% on the second day. A similar lack of recovery (to 56.0% viability) was seen when animals were only exposed during spermatogenesis in the L4 stage. These results are consistent with a role for *hasp-1* in spermatogenesis.

### HASP-1 degradation in males causes defects in mating and spermatogenesis

To independently test whether *hasp-1* was required for functional sperm, we tested whether *aid::hasp-1* males exposed to auxin starting at the L4 stage were able to produce cross progeny with hermaphrodites containing recessive visible markers (*dpy-5 unc-15*) over 48 h in the presence of auxin. Consistent with a role for *hasp-1* in spermatogenesis, cross progeny from these males was severely reduced relative to control crosses in which the plates did not contain auxin (Fig. 4E). The mating efficiency defects we observed could be due to behavioral phenotypes, so we next visualized male germlines after 24 h of auxin treatment to look for defects in spermatogenesis. Like hermaphrodites, auxin-treated male germlines did not show obvious mitotic proliferation defects. Prophase nuclei progressed normally through the karyosome stage in the proximal gonad (Fig. 4F). However, in the division zone where nuclei normally progress through both metaphase I and metaphase II divisions, and in the region containing post-meiotic spermatids (Shakes et al., 2009), we often saw elongated DAPI-stained structures. We used co-staining with anti-tubulin antibodies to better visualize nuclei undergoing divisions and saw that the elongated DAPI bodies were sometimes associated with disorganized spindles. Consistent with their reduced, but not eliminated, ability to produce cross progeny, auxin-treated males had a mix of normal and disorganized or multi-polar spindles (Fig. 4G-I). Elongated nuclei could also be found past the division zone in auxin-treated animals, throughout the region that had more uniform, circular nuclei in post-meiotic spermatids in controls. Taken together with our time-course analysis, these data suggest that *hasp-1* is required for normal spermatogenesis in both hermaphrodites and males.

### Germline HASP-1 degradation is epistatic to *plk-2* and shows synthetic embryonic viability defects with *bub-3* and *sgo-1*

**Fig. 5.**
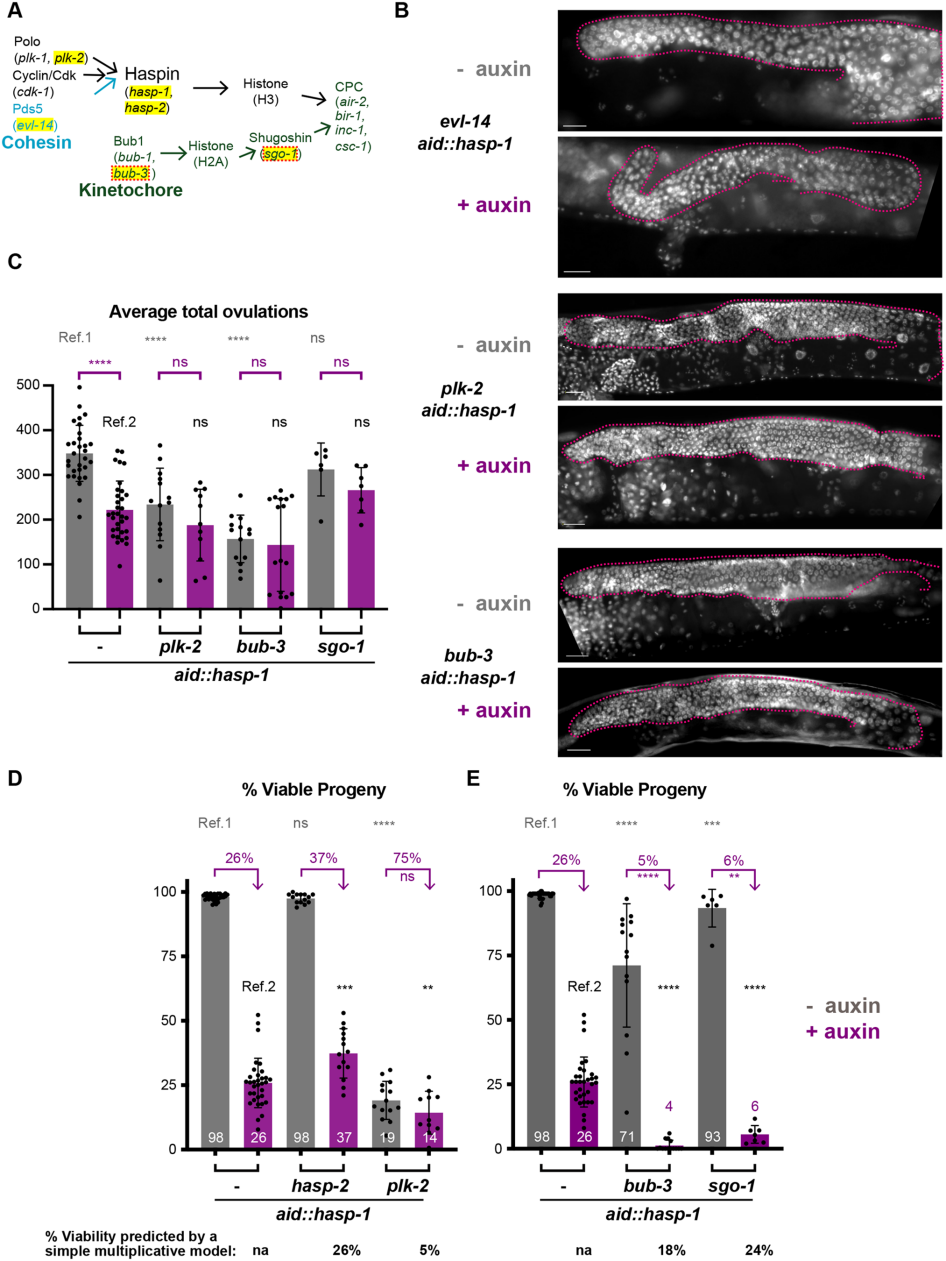
**Degradation of *hasp-1* causes synthetic lethality with Bub pathway genes.** (A) Schematic of the Haspin and Bub1 pathways of CPC recruitment. Genes investigated in this figure are highlighted in yellow. (B) Gonad nuclei in whole-mount DAPI-stained hermaphrodites of the indicated genotypes, with or without *hasp-1* depletion. The distal half of each gonad arm shown is outlined (pink). Scale bars: 20 μm. (C-E) Average total ovulations (sum of fertilized eggs and unfertilized oocytes), or % viability of whole broods. Error bars show s.d. Statistical significance indicated above gray bars for non-auxin-treated animals compares the condition to the non-auxin-treated *aid::hasp-1* animals in the wild-type background, labeled ‘Ref. 1’ (gray text), while significance indicated above purple bars refers to the comparison between the auxin treatment result in each mutant background to auxin treatment in *aid::hasp-1* animals, labeled ‘Ref. 2’ (black text). Purple text compares auxin-treated and -untreated animals of the same genotype*.* na, not applicable; ns, not significant; ****P<0.0001, ***P<0.001, **P<0.01 by Mann–Whitney test. By a simple multiplicative model, the values in purple text would all be the same and the knockdowns in mutant backgrounds would be expected to match the values shown in black below the graph.

In order to examine whether *hasp-1* interacts with known Haspin/CPC-regulators such as Pds5, Polo-like kinases or the Bub1 pathway, as shown in other organisms, we crossed *aid::hasp-1* into available mutant backgrounds that might impact HASP-1 activity and/or recruitment of the CPC (Fig. 5A). First, we examined mitosis in germline stem cells by assaying germline proliferation. We reasoned that a genetic interaction with *hasp-1* could produce a germline proliferation defect similar to the *hasp-1* deletion strain (Fig. 1); however, none of the genetic backgrounds tested produced a synthetic germline proliferation defect that was apparent cytologically (Fig. 5B, compare to Fig. 1C). Previous work had shown that strong loss-of-function alleles of the Pds5 homolog *evl-14* cause germline proliferation defects and thus have small gonads in addition to defects in cohesion and vulval development (Wang et al., 2003). We observed the small gonad phenotype in *evl-14* mutants as well as some disorganized nuclei, but this phenotype was not enhanced by HASP-1 degradation. Similarly, mutant backgrounds that have incompletely penetrant maternal-effect embryonic lethality, *plk-2* and *bub-3*, did not have any obvious reduction in gonad size or nuclei after HASP-1 degradation. As an alternative method to measure germline proliferation, we counted the total number of ovulations for individual animals as the sum of the number of eggs and unfertilized oocytes. Depletion using *aid::hasp-1* alone showed a significant reduction in ovulations, but we saw no significant reductions when HASP-1 was depleted in mutant backgrounds relative to undepleted controls (purple brackets, Fig. 5C). Thus, mitotic germline proliferation appears to be robust and less susceptible to failure in situations with low HASP-1 activity.

Next, we analyzed maternal-effect embryonic lethality in the double mutant strains to determine if genetic interactions could be measured in embryonic viability. Embryonic lethality can result from failures in meiosis as well as mitosis during embryogenesis, so this phenotype reports on the success of both mitosis and meiosis. The mutant backgrounds alone have variable embryonic viability phenotypes, so we used a simple multiplicative model to predict the viability following *aid::hasp-1* depletion in each background. If there were no genetic interaction, *aid::hasp-1* depletion would be expected to reduce viability of each background to the same extent as in the wild-type background, 26% (i.e. all purple numbers would be 26% in Fig. 5D,E), and the viability produced by the auxin-treated animals in each genotype would be 26% of the non-auxin-treated animals of that genotype (black values below the graphs, Fig. 5D,E).

The *hasp-2* or *plk-2* mutant backgrounds might further reduce the level of H3T3ph if *hasp-2* is able to substitute for *hasp-1* or if *plk-2* activates the HASP-1 protein. Consistent with the lack of phenotype in *hasp-2* mutants alone (Fig. 2), degrading HASP-1 in the *hasp-2* background did not enhance the embryonic lethality phenotype and instead caused an increase in embryonic viability relative to *aid::hasp-1* depletion in the wild-type background (37% versus 26%, Fig. 5D). The *plk-2* mutant produces a strong embryonic lethality phenotype alone (Harper et al., 2011), but HASP-1 degradation did not cause a significant further enhancement of that phenotype (14% versus 19%, Fig. 5D). A simple multiplicative model predicts that the *plk-2*, HASP-1-depleted animals would have an embryonic of 5% (19%×26%=5%) rather than the 14% viability we measured. This result suggests that *hasp-1* and *plk-2* are acting in the same pathway, which provides evidence that *plk-2* is able to activate the HASP-1 protein in *C. elegans*.

In contrast to our results with *plk-2*, we saw synthetic enhancement of the viability defect when we performed HASP-1 degradation in backgrounds with mutations that should reduce Bub1-dependent recruitment of the CPC (Fig. 5E). The *C. elegans bub-1* locus is less than a map unit away from the *hasp-1* gene, so we crossed the *aid::hasp-1* mutation into a *bub-3* deletion mutant to indirectly disrupt the function of *bub-1*. Homozygous *bub-3(ok3437)* mutants have been shown to cause a strong reduction in the level of BUB-1 protein and to result in a slight embryonic lethality phenotype and a reduced brood size (Kim et al., 2015). In our experiments, the *bub-3* mutant had a mild embryonic viability defect that was very variable (71%, s.d.=24%) likely due in part to a large variability in brood sizes (Table 2). When *aid::hasp-1* was depleted in the *bub-3* background, the viability went down almost to zero, with a majority of broods having no viable eggs (11/17). Similarly, a deletion allele of the shugoshin homolog *sgo-1* (Ferrandiz et al., 2018) had only a slight embryonic viability phenotype alone (93%), but this dropped severely when HASP-1 was degraded. In both cases, the phenotype after depletion in the mutant background was well below what would be predicted by a simple multiplicative model. These data suggest that synergistic lethality is caused by the combined knockout of both the Haspin and Bub1 pathways of CPC recruitment, confirming that both pathways are important in *C. elegans*.

**Table 2.**
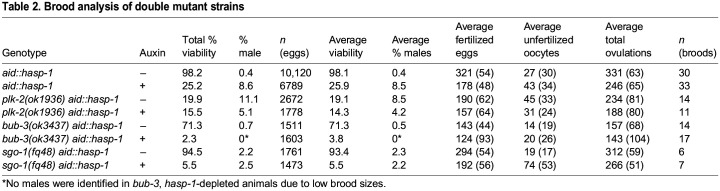
Brood analysis of double mutant strains

## DISCUSSION

### Ruling out a role for *hasp-2* in *C. elegans*

Our genetic analysis provides evidence that the Haspin-related gene *hasp-1* acts during multiple processes in *C. elegans*, including oocyte meiosis, spermatogenesis, germline stem cell proliferation and mitosis in early embryos (Figs 1 and 3). We confirmed that *hasp-1* is the main ortholog of Haspin by ruling out roles for its most closely related paralog *hasp-2* (Fig. 2). The unusual expansion of the Haspin gene family in *C. elegans* (Higgins, 2001) has left open the possibility that sub-functionalization has evolved among the different paralogs, but this does not appear to be the case for *hasp-2* in cell division. However, *hasp-2* and the other paralogs could retain the ability to phosphorylate histones, so further work will be needed to test whether these genes play important roles.

### Characterizing roles for *hasp-1* during cell division in a variety of cell types

The adult sterility we observed in animals with a *hasp-1* deletion mutation is consistent with *hasp-1* being essential for cell division because, in *C. elegans*, loss of function of cell division genes often produces sterility rather than embryonic lethality. Maternally deposited gene products are thought to be sufficient for cell division during embryonic development but then become limiting when the animals reach adulthood (O'Connell et al., 1998). Thus, the sterility phenotype of *hasp-1* deletion mutants does not alone provide evidence for other roles outside of mitosis. However, our finding that *hasp-1* deletion mutations have severe defects in germline stem cell proliferation (Fig. 1) are in contrast with a recent report that found no defect in germline proliferation in Haspin knockout mice (Soupsana et al., 2021), showing that there are differences between animals in the importance of the Haspin pathway specifically in germline stem cells, as well as more generally in mitosis.

Because the *hasp-1* deletion mutation prevented formation of a normal germline, we used the AID system to further characterize roles for *hasp-1*. We were surprised to find that germline-specific depletion of HASP-1 did not recapitulate the severe germline stem cell proliferation defect of the *hasp-1* deletion. HASP-1-depleted hermaphrodites did produce fewer overall oocytes (Table 2), which could be due to a decrease in germline stem cell proliferation, but the gonads of these animals were morphologically normal (Fig. 3A). Instead, germline HASP-1 depletion produced a variety of phenotypes that could not be observed in the deletion mutant, which are consistent with roles for *hasp-1* in spermatogenesis, oocyte meiosis and mitosis in embryos. The cell division defects we saw in embryos (Fig. 3C) were similar to those seen following RNA interference of CPC components (Kaitna et al., 2000; Romano et al., 2003; Speliotes et al., 2000), and the Him phenotype of the surviving progeny was consistent with the previously established requirement for *hasp-1* in CPC localization in oocyte meiosis (Ferrandiz et al., 2018). The low embryonic viability we measured is therefore due to a combination of meiotic and mitotic defects.

There are a number of possible explanations for the finding that the *aid::hasp-1* degradation phenotype differed from the phenotype of the deletion mutant. Depletion using *aid::hasp-1* was not able to disrupt germline stem cell proliferation as *hasp-1* deletion did. The tissue specificity of germline knockdown could be responsible for this difference, which would suggest that *hasp-1* function is required in somatic cells to support germline proliferation. However, a simpler explanation is that low levels of HASP-1 protein remaining after depletion are sufficient in proliferating stem cells but not for normal meiotic divisions. Previous work has shown that auxin-dependent degradation can occur in as little as 30 min using the same 1 mM auxin conditions used here, and that most proteins can be degraded using this treatment for 4 h (Divekar et al., 2021; Zhang et al., 2015). Our experiments, however, showed reduced residual levels of H3T3ph in mitotic cells in the gonad (Fig. 3E), and the possibility remains that there was also residual activity in oocytes that was below our detection limit.

We observed a discrepancy between the level of H3T3ph signal and our ability to detect HASP-1 protein in the gonad that could explain the different functional consequences of *aid::hasp-1* degradation. In germline stem cells, undetectable levels of HASP-1 protein in mitotic nuclei were able to generate high H3T3ph, while HASP-1 was visible in oocytes that had a similar or lower intensity of H3T3ph (Fig. 3E). This result suggests that HASP-1 may be regulated differently in mitotic and meiotic cells. A decrease in HASP-1 activity in oocytes may be a result of meiosis-specific mechanisms that support differential cohesion release, such as phosphatase activity on meiotic chromosomes (de Carvalho et al., 2008; Kaitna et al., 2002; Rogers et al., 2002; Tzur et al., 2012).

It is also possible that HASP-1's contribution to CPC recruitment is dispensable during the germline mitotic divisions even though it is required in spermatogenesis (Fig. 4) and in oocyte meiosis (Ferrandiz et al., 2018; Rogers et al., 2002). CPC recruitment by HASP-1-independent mechanisms, such as the activity of Bub1, may be able to compensate for loss of *hasp-1* more fully in germline stem cell mitosis than in meiosis or mitosis in embryos. Similarly, the maternal-effect embryonic lethality we observed upon *aid::hasp-1* degradation was not completely penetrant. The embryos that survive following maternal HASP-1 depletion presumably achieved sufficient CPC recruitment, perhaps during prometaphase of meiosis I and dependent on another recruitment pathway.

To distinguish between the effects of residual HASP-1 and differences in the importance of its activity relatively to other pathways, further work will be needed to quantify the levels of HASP-1, H3T3ph and CPC in the gonad protein more precisely. Our data suggest that HASP-1 protein levels are low in the *C. elegans* germline (Fig. 3), consistent with what has been observed for Haspin in other organisms with the exception of mouse spermatocytes (Tanaka et al., 1999). Our surprising finding that HASP-1 degradation in somatic tissue caused meiotic defects (Fig. 3B) could be explained if low HASP-1 levels make knockdown possible with small amounts of TIR1 in the germline. Our attempts to quantify H3T3ph in oocytes were complicated by technical challenges with H3T3ph antibodies. In our hands, H3T3ph antibodies must be used at low concentrations to avoid staining throughout the oocyte nucleoplasm, which we found to be present with a variety of fixation conditions (data not shown). Nucleoplasmic staining did not appear to be an artifact because it was specific to the proximal oocytes where chromosomal H3T3ph is expected, and nucleoplasmic staining was reduced when animals were exposed to auxin. The nucleoplasmic signal therefore may reflect phosphorylation of the soluble histone pool, since soluble HASP-1 may not be inhibited by the recruitment of phosphatases by LAB-1, which is localized to the chromosome axis (de Carvalho et al., 2008; Ferrandiz et al., 2018).

### Genetic interactions within and between the Haspin and Bub1 pathways

Combining AID-based degradation of HASP-1 with other mutant backgrounds allowed us to support models for the activation of HASP-1 protein and the recruitment of the CPC. The parallel roles of Haspin and Bub1 pathways in CPC recruitment were supported by genetic interactions in *Schizosaccharomyces pombe*, and we set out to take a similar approach (Yamagishi et al., 2010). We found that HASP-1 depletion in the *plk-2* background caused a less severe phenotype than would have been expected from the phenotypes caused by the loss of function of each alone, which is consistent with *hasp-1* and *plk-2* acting in the same pathway. This conclusion is also based on evidence from *Xenopus* and human cells that Plk1 phosphorylates Haspin to activate it (Ghenoiu et al., 2013; Zhou et al., 2014). Our results provide the most direct evidence to date that *plk-2* is able to activate the HASP-1 protein in *C. elegans* and that this regulatory mechanism is conserved in this model system.

In contrast to our results with *hasp-1* and *plk-2*, we saw synthetic interactions between *hasp-1* and the Bub1 pathway components *bub-3* and *sgo-1*. These results were somewhat surprising because previous work in *C. elegans* showed that depletion of *hasp-1* using a similar degron allele completely removed CPC localization in diakinesis oocytes and that *sgo-1* deletion did not affect CPC recruitment (Ferrandiz et al., 2018). However, although the Bub1 pathway is therefore not active in recruitment of the CPC in diakinesis, our data provide evidence that there is still a role for the Bub1 pathway in CPC recruitment in oocytes, perhaps later in meiosis after BUB-1 protein is recruited to kinetochores and the ring that forms at the mid-bivalent (Dumont et al., 2010). Previous work on the function of meiotic axis proteins has shown that the CPC can still be rapidly recruited to chromosomes in prometaphase in mutants that fail to recruit the CPC in diakinesis (Sato-Carlton et al., 2018). Thus, further work on CPC recruitment in *C. elegans* will help tease out the robust mechanisms that recruit this important complex.

## MATERIALS AND METHODS

### Worm strains and maintenance

All worms were maintained on MyoB plates seeded with OP50 bacteria. All crosses were conducted at 20°C and genotypes were monitored by PCR. The deletion allele of *hasp-1* was obtained from the Japanese Bioresource Center (Gengyo-Ando and Mitani, 2000). Worm strains used in this study were as follows: DJW05 – *hasp-2(pdx1)* I; DJW31 – *hasp-1*(*pdx3* [*3xflag::aid::hasp-1*]) I; i*eSi38* [*sun-1p::TIR1::mRuby::sun-1 3'UTR+Cbr-unc-119(+)*] IV; DJW32 – *hasp-1*(*pdx3*) I; *ieSi57* [*eft-3p::TIR1::mRuby::unc-54* 3'UTR+Cbr-*unc-119*(+)] II; DJW36 – *hasp-2(pdx1) hasp-1*(*pdx3*) I; *ieSi38* IV; DJW39 – *hasp-1*(pdx3) I; *bub-3(ok3437)* II; *ieSi38* IV; DJW42 – *plk-2(ok1936) hasp-1*(*pdx3)* I; *ieSi38* IV; DJW47 – *hasp-1*(pdx3) I; *ieSi38 sgo-1(fq48)* IV; DJW49 – *hasp-1*(pdx3) I; *unc-36(e251) evl-14(ar112)/dpy-17(e164) unc-32(e189)* III; *ieSi38* IV; DJW64 – *hasp-1*(*tm3858*)/*tmC18* [*dpy-5(tmIs1236*)] I; *ieSi38 ltIs37 [pAA64; pie-1/mCherry::his-58; unc-119*(+)] IV; *qIs56 [lag-2p::GFP+unc-119(+)*] V; DR429 – *dpy-5(e61) unc-15(e73)* I; N2 – wild type.

### Genome editing

New alleles were generated by injection of Cas9-gRNA RNP complexes using *dpy-10* Co-CRISPR to enrich for edited progeny (Arribere et al., 2014; Paix et al., 2015). Cas9-NLS protein was complexed *in vitro* with equimolar quantities of duplexed tracrRNA and target-specific crRNAs. All RNP components were purchased from IDT. Repair templates were single-stranded Ultramer or Megamer oligonucleotides purchased from IDT. Each 10 μl of injection mixture contained: 16 mM [Cas9 protein+gRNA] (target gene+*dpy-10*), plus 0.5-1.5 μM repair templates. Injected animals were transferred to individual plates, incubated at 20 °C, and screened for F1 Rol or Dpy progeny.

Disruption of the *hasp-2* gene was accomplished by inserting the sequence GCTAG after the first base in the 14th codon, introducing a stop codon, a frameshift and an Nhe I restriction site. The *3xFLAG::aid::hasp-1* allele was generated by inserting a 70 amino acid tag [based on those used in (Zhang et al., 2018)] after the 3rd codon of *hasp-1*, full sequence shown below:

3xFLAG::AID tag (with GAGS spacer in between the FLAG and AID, lower case)

GATTATAAAGACCATGATGGAGACTATAAGGATCACGATATTGATTACAAAGACGATGATGATAAAggagccggatctCCTAAAGATCCAGCCAAACCTCCGGCCAAGGCACAAGTTGTGGGATGGCCACCGGTGAGATCATACCGGAAGAACGTGATGGTTTCCTGCCAAAAATCAAGCGGTGGCCCGGAGGCGGCGGCGTTCGTGAAG.

### Protein sequence analysis

Wild-type *C. elegans* gene sequences were obtained in FASTA format from Wormbase (www.wormbase.org). For the HASP-1 and HASP-2 protein sequences, the conceptual translations from Wormbase were used, while homologous human, mouse, *S. pombe* and *Saccharomyces cerevisiae* sequences were obtained from the NCBI database (https://www.ncbi.nlm.nih.gov/protein/). Accession numbers are as follows: human Haspin (NP_114171.2), mouse (BAB00640.1), *S. pombe* (CAB16874.1), *Drosophila melanogaster* (NP_001015349.2). All alignments were done using Clustal Omega (Sievers et al., 2011) and visualized using Jalview (Waterhouse et al., 2009).

### Embryonic viability, male frequency and brood size assays

Percent viability, male frequency and brood size of self progeny were measured by placing single L4 hermaphrodites onto 60 mm plates at 20°C and transferring worms to a new plate every 24 h until they stopped laying. Dead eggs were scored 24 h after the mother had been removed and the number and sex of F1 were scored after they reached the adult or L4 stages. The embryonic viability for each worm was calculated by dividing the total number of progeny by the total number of worms and dead eggs laid, while percent males was the number of males divided by the total progeny. In all graphs, the % viability shown is the average for all worms for which the entire brood was counted. Brood size was the total number of worms and dead embryos, and the total ovulations was the sum of worms, eggs and unfertilized oocytes.

### Male mating assays

Mating efficiency was measured by placing six *dpy-5(e61) unc-15(e73)* L4 hermaphrodites on 60 mm plates (with or without 1 mM auxin) with six L4 males. Males and hermaphrodites were allowed to mate for 48 h at 20°C. Males were removed from the plate, and hermaphrodites were transferred to new plates. Hermaphrodites were then transferred to new plates every 24 h until egg laying stopped. Plates were scored at 72 h of age. The number of wild-type (outcross) and Dpy Unc (self) offspring were counted for each plate, and male mating efficiency was calculated by dividing the number of outcross offspring by the total number of offspring.

### Western blotting

Adult worms were picked onto unseeded plates to remove bacteria, collected off plates with M9, excess M9 was removed, and an equal volume of 2× SDS sample buffer was added before boiling for 10 min. The final volume and number of worms in each sample were measured so that each lane of the gel was loaded with lysate of approximately 100 animals. Blots were blocked with 4% bovine serum albumin and probed with anti-phospho-Histone H3 (Thr3) (EMD Millipore 07-424) diluted 1:5000, anti-FLAG (Sigma-Aldrich F1804) diluted 1:200 and anti-α-tubulin (Sigma-Aldrich T6199) diluted 1:5000. HRP secondary antibodies (diluted 1:5000) and chemiluminescent substrate were purchased from LI-COR and imaged using a C-Digit 3600 (LI-COR).

### Immunofluorescence

Dissected gonads were fixed in 2% formaldehyde in egg buffer containing 0.1% Tween 20 for 5 min, freeze cracked into cold methanol and transferred to PBS+0.1% Tween 20 at room temperature. Primary antibodies used were anti-phospho-Histone H3 (Thr3) (EMD Millipore 07-424) diluted 1:10,000, anti-FLAG (Sigma-Aldrich F1804) diluted 1:500, anti-α-tubulin (Sigma-Aldrich T6199) diluted 1:1000, anti-HTP-3 [gift from A. F. Dernburg (MacQueen et al., 2005)] diluted 1:500. Secondary antibodies were purchased from Jackson ImmunoResearch Laboratories. DAPI staining was done using 0.5 μg/ml in PBS on both dissected gonads or whole-mount animals after cold methanol fixation. Samples were mounted in Prolong Gold mounting medium (Millipore Sigma).

### Microscopy

Confocal imaging was performed on a Diskovery spinning disk confocal system (Andor) mounted on a Nikon Eclipse Ti microscope with a 60×1.4 NA objective. Epifluorescence and differential interference contrast (DIC) (Nomarski) imaging were performed on an Olympus BX60 microscope with a Plan Apo 60×1.42 NA objective.
